# Merkel cell carcinoma occurring in a black woman: a case report

**DOI:** 10.1186/s13256-016-1189-8

**Published:** 2017-01-31

**Authors:** Selma Kadiri, Abdellah Aissa, Soufiane Berhili, Mouna Khmou, Sanaa Elmajjaoui, Tayeb Kebdani, Basma El Khannoussi, Hanan Elkacemi, Noureddine Benjaafar

**Affiliations:** 1 Department of Radiotherapy, National Institute of Oncology, Rabat, Morocco; 2 Department of Cytopathology, National Institute of Oncology, Rabat, Morocco

**Keywords:** Merkel cell carcinoma, Dark skin, Elbow, Radiotherapy

## Abstract

**Background:**

Merkel cell carcinoma is a rare, very aggressive neuroectodermal tumor of the skin. It is typically located on sun-exposed skin and frequently found in white men aged between 70 and 80 years.

**Case presentation:**

We report a case of a 58-year-old black woman diagnosed with Merkel cell carcinoma of the posterior face of the right elbow. She had biopsy excision and was lost to follow-up. Four months later, she presented with recurrent disease on the inferior third of the right arm with three ipsilateral axillary lymph node metastases. Amputation of the right arm and ipsilateral axillary lymph node dissection were performed, followed by adjuvant radiotherapy. Six months later, the patient died as a result of respiratory failure caused by lung metastasis. To the best of our knowledge, no specific studies have been done comparing the course and the characteristics of Merkel cell carcinoma in white and black populations, and no similar case has been reported in the literature.

**Conclusions:**

The Merkel cell carcinoma is very rare in black people. As described elsewhere in the literature, our patient had a poor outcome despite radical management. To date, to the best of our knowledge, there has been no comparison of the prognosis of this tumor in white and black populations.

## Background

Merkel cell carcinoma (MCC) is a rare and very aggressive neuroendocrine tumor of the skin [1]. It affects adults with light skin types in the seventh decade of life and has a high rate of local recurrence and regional lymph node metastasis [2]. MCC was first described in 1972 by Toker [3]. It seems to arise from the basal layer of the epidermis where specific cells—the Merkel cells—are located [4]. This hypothesis is controversial, and recent observations suggest that these tumors originate from an immature totipotential dermal stem cell that acquires neuroendocrine features during malignant transformation [5].

We report a rare case of an epidemiological presentation of MCC occurring in the right elbow of a black woman. We also try to show through this case if there are differences in prognosis between MCC occurring in white and black people.

## Case presentation

**Fig. 1:**
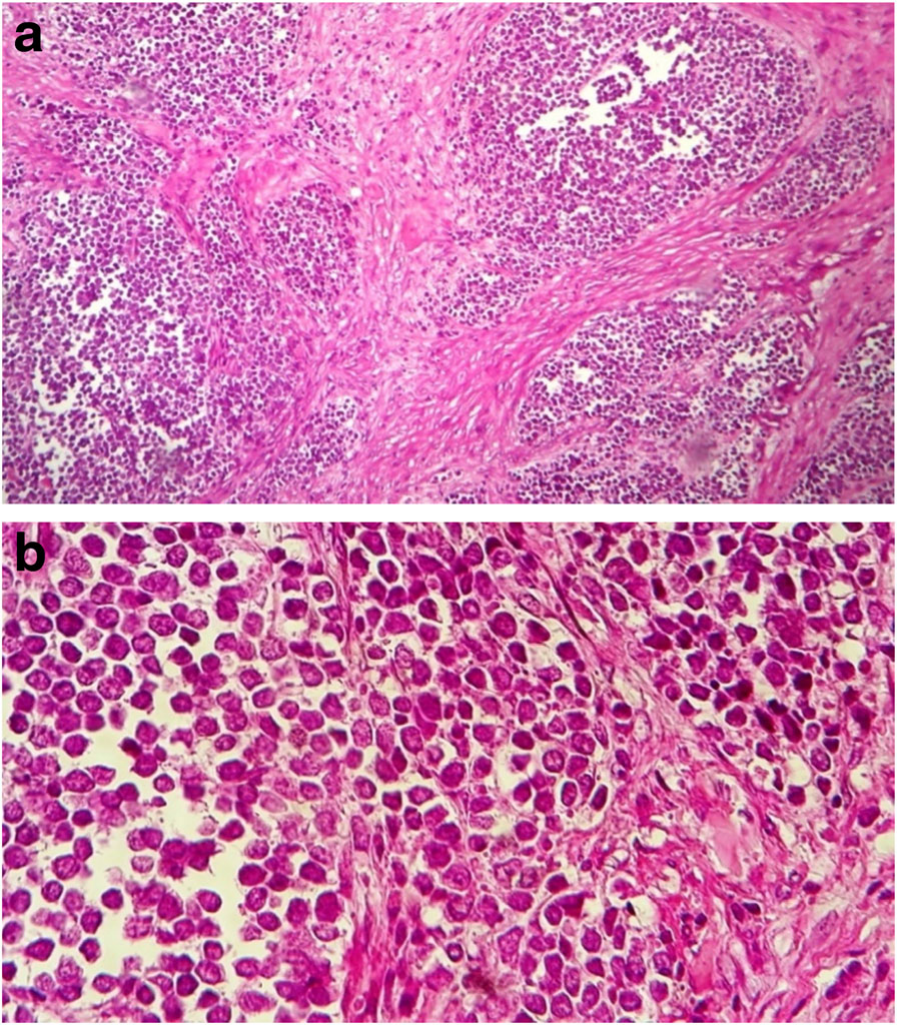
**a** Cutaneous neuroendocrine carcinoma. Typical low-power view of a small blue-cell tumor (hematoxylin and eosin stain, original magnification ×40). **b** Microphotograph showing pale-staining, small, round cell proliferation containing tiny nucleoli (hematoxylin and eosin stain, original magnification ×40)

**Fig. 2:**
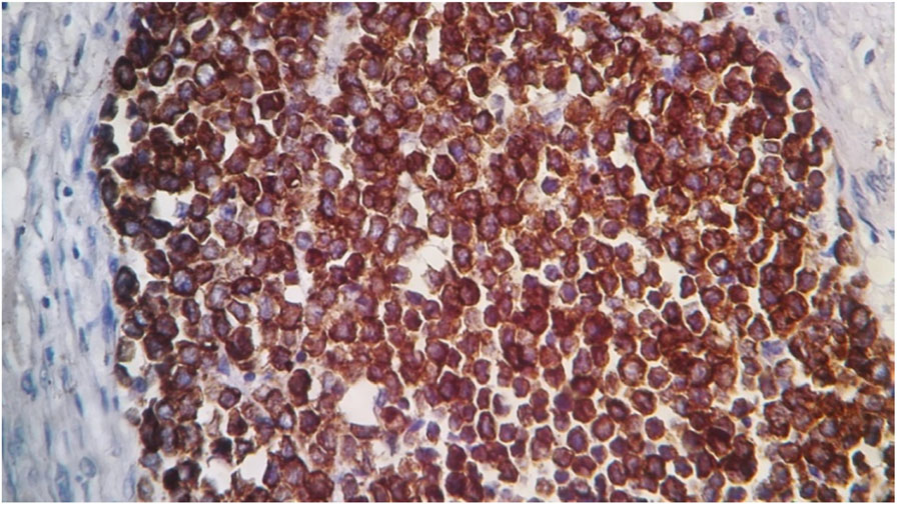
High-power view of the tumor showing strong expression of cytokeratin 20 (original magnification ×40)

**Fig. 3:**
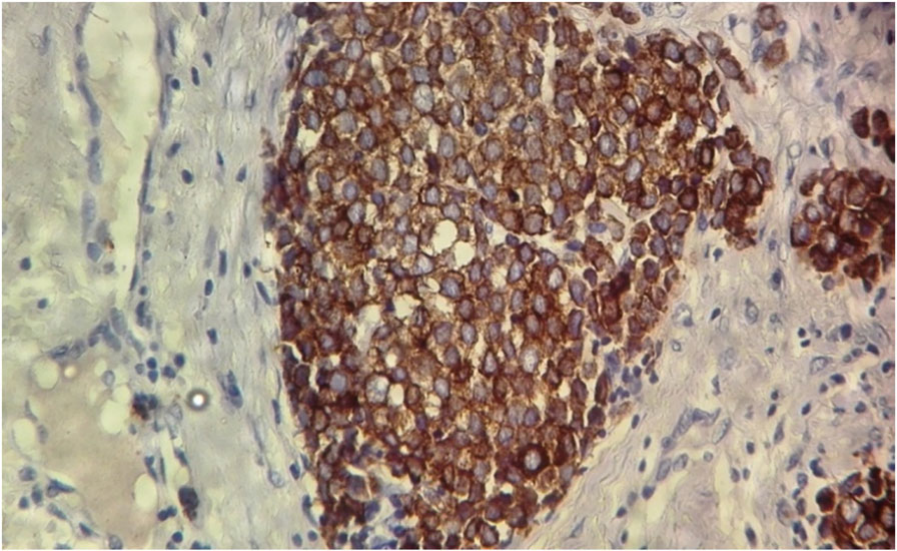
High-power view of the tumor showing strong expression of synaptophysin (original magnification ×40)

**Fig. 4:**
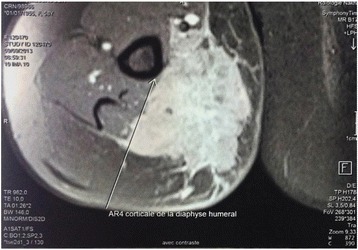
Contrast-enhanced, T1-weighted axial magnetic resonance imaging scan showing the process of the soft parts on the right arm

A 58-year-old African black woman with a medical history of diabetes presented to our institute with a rapidly growing skin lesion that was initially a small, round, painless nodule on the posterior face of the right elbow. In a physical examination at the time of admission, a 50 × 30-mm purple lesion with an irregular surface on the posterior face of the right elbow and extending to the forearm was found. No ipsilateral axillary lymph nodes were found. The patient had stage II disease. A biopsy excision was performed. The pathological examination showed a proliferation of round, small cells (Fig. 1a, b). An immunohistochemical study using specific staining techniques confirmed the diagnosis of MCC and, more specifically, cytokeratin 20 (CK20) expression, which is often evident as a paranuclear globule (Fig. 2); synaptophysin (Fig. 3); CD99; neuron-specific enolase; and negativity of the melanocytic and muscular markers. The patient was lost to follow-up and consulted us 4 months later. The physical examination done at that time revealed a large inflammatory lesion of 100 × 50 mm on the third inferior side of the right arm with three ipsilateral axillary lymph nodes. A magnetic resonance imaging (MRI) scan of the right arm showed a process of the soft parts of 103 × 63 × 50 mm with three metastatic ipsilateral axillary lymph nodes (Fig. 4).

A biopsy of one of the axillary lymph nodes confirmed the diagnosis of MCC. Computed tomography (CT) of the chest, abdomen, and pelvis showed no distant metastasis. The treatment planning consisted of surgical amputation of the right arm with ipsilateral axillary lymph node dissection. A histopathologic examination revealed negative surgical margins. The margins were found to be microscopically uninvolved by carcinoma, with the closest margin at 4 mm (deep margin). Of 31 excised lymph nodes, 13 that were metastatic with capsular rupture were found.

**Fig. 5:**
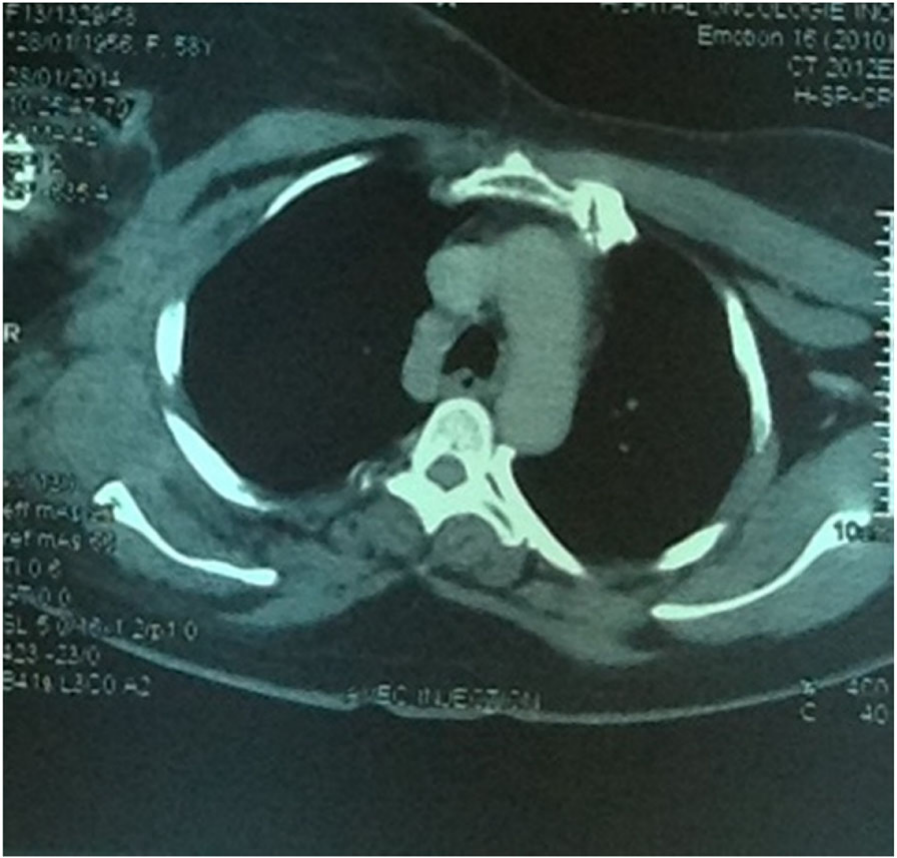
Computed tomography slice showing a mass in the right axillary and subscapularis regions

The patient was referred to our radiation therapy department. At that admission, the examination revealed a clean amputation stump and no axillary lymphadenopathy, but the patient had a fixed mass in the right axilla. A CT scan showed a process in the right axillary region extending to the right subscapularis fossa (Fig. 5). No surgical treatment was possible. A decision was made to perform radiation therapy of the right axillary and subscapularis regions. The total dose was 66 Gy with a conventional fractionation of 2 Gy per fraction in 33 sessions, 5 days per week, in the right axillary and subscapular areas in two oblique fields: anterior right and posterior left fields. The patient had grade 2 radiodermatitis as the only acute toxicity of radiotherapy.

Six months later, the patient presented with dyspnea caused by lung metastasis, and she died 1 month afterward as a result of respiratory failure.

## Discussion

MCCs are rare tumors of the skin. According to National Cancer Institute Surveillance, Epidemiology, and End Results Program data, the estimated annual incidence in 2006 was 0.6 per 100,000 persons [6]. In Australia and New Zealand, higher incidence rates are reported [7]. These tumors occur mostly in the white population (94%) [8, 9]. Males develop MCC more often than females, with incidence rates of 0.41 and 0.18 cases per 100,000 person-years, respectively [6]. MCC is typically seen in the elderly, with mean ages at diagnosis of 74 years for men and 76 years for women [6]. Our patient was a 58-year-old woman.

MCCs often occur on sun-exposed regions of the skin [8, 9]. The etiology is largely uncertain, though there is some evidence that ultraviolet radiation and immune system depression are important risk factors. More recently, some studies have shown the role of the Merkel cell polyomavirus (MCV) group in the development of this tumor [8, 10]. MCV has been isolated in up to 80% of the MCC tissue analyzed [11].

MCC presents as a painless, purple lump on the skin, sometimes ulcerated. It can be multifocal [1]. The head and neck are the most common primary sites (48%), followed by the upper limbs (19%), lower limbs (16%), and trunk (11%) [12]. Most patients (73%) present with localized disease (stages I–II); 23% have regional disease (stage III), and 4% have stage IV metastatic disease [12].

The histological presentation of MCC is small, round, blue-cell tumors and need to be distinguished from lymphoma, melanoma, sarcoma, and metastatic skin deposits from other neuroendocrine carcinomas, in particular small cell lung carcinoma. Immunohistochemical study characteristics of neuroendocrine carcinoma of the skin is low-molecular-weight (CAM 5.2) keratin, and more specifically CK20 expression, which is often evident by paranuclear immunostaining [13]. CK7 is characteristically negative in MCC and may be positive in small cell lung carcinoma [13]. Neuroendocrine markers (neurofilament protein, chromogranin, and synaptophysin) are frequently expressed in MCC tumors [14]. The tumor cells are negative for leukocyte common antigen, HMB-45, Melan-A, desmin, and myogenin. A protocol of examination of specimens from patients with MCC has been developed to assist pathologists in providing clinically relevant information [15, 16].

Common sites of MCC metastasis include distant lymph nodes (60%), distant skin (30%), lung (23%), central nervous system (60%), and bone (15%) [12]. CT and MRI scans are obtained to evaluate MCC and for treatment planning, but there is no accepted imaging algorithm [8]. Recently, however, some studies have shown that fluorodeoxyglucose-positron emission tomography is a highly sensitive modality for MCC evaluation before and after treatment [5].

The standard treatment of the primary tumor remains surgical. Removal by wide local excision is typical, with some surgeons also using Mohs micrographic surgery to ensure adequate clear margins at excision [17]. Lymph node dissection and primary nodal radiotherapy are options for treating clinically node-negative disease. For clinically palpable regional disease, lymph node dissection is recommended with consideration of adjuvant radiation therapy. In recent studies, the role of sentinel lymph node biopsy in guiding adjuvant treatment has been established [18]. Adjuvant radiotherapy doses ranged from 45 to 66 Gy, depending on the techniques used and the presence of microscopic or gross disease after surgery. The role of neoadjuvant and adjuvant chemotherapy is not clear and should be analyzed in more studies [8].

Prognostic factors are primary tumor size up to 2 cm and a high mitotic index, which may contribute to a higher risk of locoregional recurrence [19, 20]. An increasing number of metastatic nodes is associated with significantly worse overall survival [8, 21]. Five-year survival is 57% for localized disease, 39% for regional disease, and 18% for metastatic disease [19, 22]. Recurrence rates of MCC are high, with most occurring within 2 years (often earlier) and increasing with higher stage at presentation [23, 24].

In our patient, the tumor size was up to 2 cm, and she presented with locoregional recurrence very early (4 months). She died after lung metastasis only 6 months after the end of treatment. To the best of our knowledge, there are no studies in the literature in which researchers analyzed the impact of skin color and geographic or ethnic origin on the prognosis of MCC. As in the other nonmelanoma skin cancers (basal and squamous cell carcinoma), routine skin scanning and dermatological examination should be done in the elderly population to provide early diagnosis and therapeutic care [25].

## Conclusions

MCC presents rarely in dark-skinned people, which suggests a protective role of their skin pigmentation. As seen in our patient and as reported in the literature, MCC has a poor outcome. However, more studies should be done to analyze the difference in prognosis between light- and dark-skinned populations. The high rate of local and distant recurrence of MCC despite radical management and a multidisciplinary approach deserve more research on the role of systemic treatment.

## Abbreviations

CK: Cytokeratin
CT: Computed tomography
MCC: Merkel cell carcinoma
MCV: Merkel cell polyomavirus
MRI: Magnetic resonance imaging
